# iSPHYNCS: Unsupervised Clustering in Questionnaires and Metadata Reveals Distinct Subtypes in the Narcolepsy Borderland

**DOI:** 10.1111/jsr.70294

**Published:** 2026-02-09

**Authors:** Rafael Morand, Livia Fregolente, Julia van der Meer, Elena S. Wenz, Annina Helmy, Lorenzo Brigato, Jan D. Warncke, Kseniia Zub, Ramin Khatami, Zhongxing Zhang, Sigrid von Manitius, Silvia Miano, Jens Acker, Mathias Strub, Ulf Kallweit, Gert Jan Lammers, Athina Tzovara, Claudio L. A. Bassetti, Stavroula Mougiakakou, Markus H. Schmidt

**Affiliations:** ^1^ Sleep‐Wake Epilepsy Center, NeuroTec, Department of Neurology, Inselspital, Bern University Hospital University of Bern Bern Switzerland; ^2^ Center for Experimental Neurology, Sleep Wake Epilepsy Center, NeuroTec, Department of Neurology, Inselspital, Bern University Hospital University of Bern Bern Switzerland; ^3^ ARTORG Center for Biomedical Engineering Research University of Bern Bern Switzerland; ^4^ Graduate School for Cellular and Biomedical Sciences University of Bern Bern Switzerland; ^5^ Graduate School for Health Sciences University of Bern Bern Switzerland; ^6^ Institute of Computer Science University of Bern Bern Switzerland; ^7^ Clinic Barmelweid Center for Sleep Medicine and Sleep Research Barmelweid Switzerland; ^8^ Department of Neurology Health Ostschweiz (HOCH), Kantonsspital St. Gallen St St. Gallen Switzerland; ^9^ Sleep and Epilepsy Center, Neurocenter of Southern Switzerland, Regional Hospital (EOC) of Lugano Lugano Switzerland; ^10^ ZurzachCare, Clinic for Sleep Medicine Bad Zurzach Switzerland; ^11^ Centre for Sleep Medicine Basel Basel Switzerland; ^12^ Center for Narcolepsy and Hypersomnias, Professorship for Narcolepsy and Hypersomnolence Research, Department of Medicine University Witten/Herdecke Witten Germany; ^13^ Sleep Wake Centre SEIN, Stichting Epilepsie Instellingen Nederland Heemstede the Netherlands; ^14^ Department of Neurology, Leiden University Medical Centre Leiden the Netherlands

**Keywords:** central disorders of hypersomnolence, clustering analysis, fatigue, iSPHYNCS, narcolepsy, psychiatric burden, sleepiness

## Abstract

The international Swiss Primary Hypersomnolence and Narcolepsy Cohort Study (iSPHYNCS) is a multicentre study aimed at identifying novel biomarkers for central disorders of hypersomnolence (CDH). We analysed questionnaires and metadata to uncover distinct clusters of participants and explore phenotypic variability within CDH. Data were collected from 227 patients with CDH and 33 healthy controls. Participants completed validated clinical questionnaires and study‐specific questions addressing CDH‐related symptoms such as excessive daytime sleepiness, fatigue, cataplexy, disrupted sleep, and sleep paralysis. Demographic metadata (age, gender, BMI) were included. After excluding participants with missing over 30% of data (*n* = 40), missing values were imputed using a multiple random forest algorithm. A robust clustering pipeline was employed: (1) random sampling of 60% of the dataset, (2) dimensionality reduction via UMAP, (3) K‐means clustering, and (4) consensus clustering across 500 iterations. Post hoc analysis was performed to identify biomarkers in data not used for clustering. We identified four distinct clusters. One predominantly comprised healthy controls, while another primarily contained individuals with narcolepsy type 1 (NT1). Two clusters represented predominantly the narcolepsy borderland group (NBL), with one distinctly characterised by higher symptom severity and psychiatric comorbidities. The clustering pipeline produced reproducible results, with the NT1 and healthy control clusters serving as internal validation. The differentiation between the two NBL clusters aligns with prior studies, suggesting a possible NBL subtype marked by increased fatigue and psychiatric comorbidities. These findings emphasise the phenotypic heterogeneity of CDH and the potential for cluster‐based approaches in management.

**Trial Registration:** ClinicalTrials.gov identifier: NCT04330963.

## Graphical abstract

Study overview and clustering results in the iSPHYNCS cohort. Participant demographics are summarized in a baseline table. Questionnaire data and metadata were processed through an unsupervised clustering pipeline. A Sankey plot illustrates individual assignment across clusters by diagnostic group. The barcode plot highlights the most discriminative clinical features characterizing each cluster, emphasizing heterogeneity in symptom burden and functional impairment across central disorders of hypersomnolence.

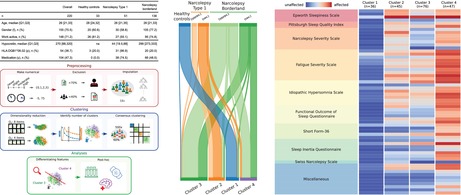

## 1 Introduction

Central disorders of hypersomnolence (CDH) represent a group of diseases characterised by an overwhelming and irrepressible need for sleep that impacts daily functions. These disorders are currently classified according to the International Classification of Sleep Disorders, third edition (ICSD‐3) (Sateia 2014). Nevertheless, due to often overlapping and ambiguous diagnostic criteria, there have been numerous recent efforts attempting to reassess and refine the current classification system (Lammers et al. 2020; Dietmann, Wenz, van der Meer, et al. 2021). While experts agree that narcolepsy type 1 (NT1) can be reliably differentiated by the presence of cataplexy and absence or low levels of hypocretin in the cerebrospinal fluid (Bassetti et al. 2019), other CDH disorders from the so‐called narcolepsy borderland (NBL), such as narcolepsy type 2 and idiopathic hypersomnia, rely heavily on subjective assessments and lack pathophysiological specificity (Zhang et al. 2022).

Recent advancements in data science, particularly machine learning techniques, have introduced new possibilities for phenotyping sleep–wake disorders based on clinical and biological data (Zinchuk and Yaggi 2020; Venkatnarayan et al. 2023). Clustering algorithms, for instance, are designed to group patients into phenotypes that exhibit minimal intra‐group variability and well‐defined inter‐group differences (Bailly et al. 2016). In the context of CDH, unsupervised clustering methods have shown promise in reclassifying patients into phenotypes that may be more clinically meaningful than traditional observations (Gool et al. 2022; Aellen et al. 2024). More specifically, identified clusters distinguished patients within the NBL based on the presence or absence of sleep drunkenness (Gool et al. 2022), as well as demographical and electrophysiological markers (Aellen et al. 2024).

A further study employing unsupervised clustering approaches in patients diagnosed with hypersomnolence according to the fifth edition of the Diagnostic and Statistical Manual of Mental Disorders (DSM‐5) identified clusters differentiated by variables such as the presence of depression and other clinical features (Cook et al. 2019). These findings underscore the importance of considering psychiatric comorbidities, which have been shown to exacerbate the impact of CDH and contribute to a vicious cycle of fatigue, sleepiness and reduced quality of life (Aktan Suzgun et al. 2024).

While unsupervised learning techniques have provided promise in refining diagnostic categories for CDH, it remains unclear whether these phenotypes are generalizable and applicable in other populations. Moreover, previous observations have relied on the presence of electrophysiological and biological data without a control group for comparison. Therefore, the aim of this study was to apply unsupervised clustering techniques to reclassify patients with CDH based on a comprehensive set of clinical data, including a healthy control group. To achieve this, we utilised the well‐characterised international Swiss Primary Hypersomnolence and Narcolepsy Cohort Study (iSPHYNCS) (Dietmann, Wenz, van der Meer, et al. 2021), a multicentre prospective cohort study involving collaboration across 10 centres in three European countries. We hypothesised that by using data from questionnaires and metadata, unsupervised clustering would (a) result in a clear separation of healthy controls from patient groups, (b) result in a clear separation of individuals diagnosed with NT1 from patients with other CDH without cataplexy, and (c) group individuals from the NBL into multiple clusters according to the perceived severity of symptoms. We are confident that the findings from our cluster analysis will provide a refined foundation for future therapeutic frameworks of the NBL.

## 2 Methods

### 2.1 Study Design and Setting

This study was conducted across 10 sleep centres located in three European countries—Switzerland, Germany, and the Netherlands—as part of the prospective international Swiss Primary Hypersomnolence and Narcolepsy Cohort Study (iSPHYNCS). The iSPHYNCS is a multicentre study aiming at investigating the phenotypes and clinical characteristics of individuals with central disorders of hypersomnolence. The complete study protocol has been previously published (Dietmann, Wenz, van der Meer, et al. 2021). This analysis uses data from participants enrolled at the following sleep clinics and study sites (listed in alphabetical order): Bad Zurzach (Switzerland); Barmelweid (Switzerland); Basel (Switzerland); Bern (Switzerland); Heemstede (the Nederlands); Lugano (Switzerland); St. Gallen (Switzerland); and Witten (Germany).

### 2.2 Participants

We included individuals aged 16–70 years who presented to the participating sleep centres with subjective complaints of excessive daytime sleepiness (EDS) and/or hypersomnolence. Patients included in this analysis were consecutively recruited into the study between September 2020 and October 2024 and needed to have completed at least 70% of the study's questionnaires. Eligible participants were fluent speakers of German, French, Dutch, or Italian and provided informed written consent prior to participation. Exclusion criteria included the presence of other sleep disorders, neurological and severe or unstable psychiatric conditions, or systemic diseases that could more likely explain their EDS or hypersomnolence. Healthy controls were recruited from local advertising and excluded if showing signs of EDS or significant SDB.

Participants were provisionally categorised based on the International Classification of Sleep Disorders—Third Edition (ICSD‐3) (American Academy of Sleep Medicine 2014) into three groups: NT1, healthy controls (HC), and the broader narcolepsy borderland (NBL). The NBL group encompassed diagnoses such as narcolepsy type 2, idiopathic hypersomnia, insufficient sleep syndrome and hypersomnia associated with a psychiatric disorder. We also included patients who did not meet criteria for a specific CDH; these patients were classified as excessive daytime sleepiness not otherwise specified.

Reviewed diagnoses were established by a panel of at least two clinician experts using ICSD‐3 criteria. For NT1, the diagnosis was also considered as reviewed if available cerebrospinal fluid hypocretin‐1 levels were below 110 pg/mL (Bassetti et al. 2019). In the other cases, provisional diagnoses assessed by the treating physician were considered for analysis as unreviewed.

### 2.3 Data Collection

Data were collected prospectively using standardised questionnaires and a systematic interview. In December 2022, the study expanded to include international collaborators, transitioning from SPHYNCS to iSPHYNCS, and the range of assessment tools was adjusted. For instance, the Mini International Neuropsychiatric Interview (MINI) (Sheehan et al. 1998) and the Beck depression inventory‐II (Beck et al. 1996) were replaced with the hospital anxiety and depression scale (HADS) (Zigmond and Snaith 1983).

This analysis includes only responses obtained at inclusion and for questions completed by at least 60% of the cohort. The nine questionnaires included in the clustering pipeline, and for which licences have been obtained, are: Epworth sleepiness scale (ESS) (Johns 1991), fatigue severity scale (FSS) (Valko et al. 2008), narcolepsy severity scale (NSS) (Dauvilliers et al. 2017), Swiss narcolepsy scale (SNS) (Bargiotas et al. 2019), Pittsburgh sleep quality index (PSQI) (Buysse et al. 1989), idiopathic hypersomnia severity scale (IHSS) (Dauvilliers et al. 2019), sleep inertia questionnaire (subset of SIQ) (Kanady and Harvey 2015), 36‐item short form survey instrument (subset of SF36) (Hays et al. 1993) and functional outcome of sleep questionnaire (FOSQ) (Weaver et al. 1997). These instruments, together with metadata variables (age, gender, body mass index, and work status) captured a broad range of subjective clinical variables, including sleep quality, hypersomnolence severity, and functional impairment. To minimise potential treatment‐related confounding, participants already receiving therapy were instructed to fill out all questionnaires according to their status immediately prior to treatment initiation. Diagnostic labels were not used for the clustering process. An extensive description of the questionnaires is provided in the Supporting Information S1.

### 2.4 Unsupervised Clustering

**FIGURE 1:**
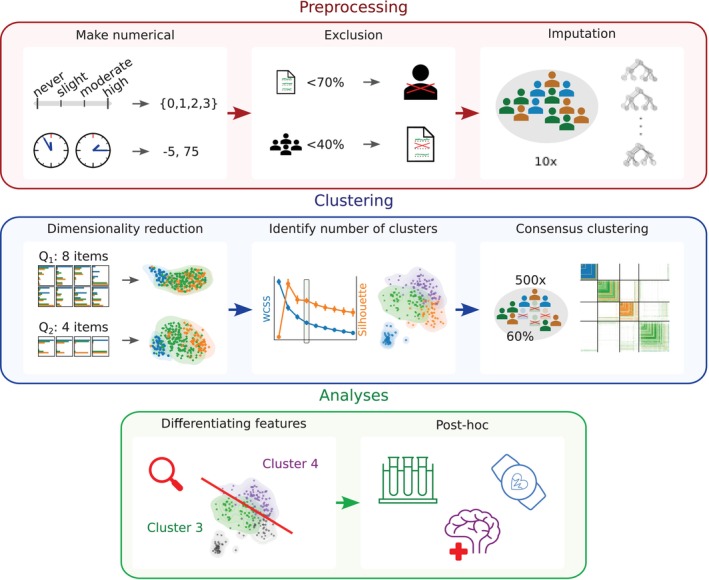
Schematic of the preprocessing and clustering pipeline, and the subsequent analyses.

We employed an unsupervised clustering pipeline to identify distinct phenotypic subgroups within the study cohort (Figure 1). A detailed description of this approach is provided in the Supporting Information S2.

For the preprocessing, we encoded categorical data to be interpreted as numerical values, and timestamps were transformed into minutes relative to midnight. Then, we excluded individuals if they had answered less than 70% of the questions. In addition, we excluded variables if they were answered by less than 40% of the study participants. For imputation, we performed multiple imputation with 10 iterations using a random forest imputer (GitHub repository of Missingpy, accessed 25.01.2024) (Aellen et al. 2024). To avoid biasing the imputed values towards the large NBL group, we processed the three cohorts separately (HC, NT1, NBL). For normalisation, we scaled each variable to a range from −1 to 1 across all cohorts.

Reducing the dimensions for all questionnaires to a common fixed size is beneficial as it gives equal weights to all questionnaires in the clustering algorithm. Hence, we reduced the dimensionality using UMAP (McInnes et al. 2018) on each questionnaire individually to compress the N‐dimensional questionnaires into two dimensions, where N denotes the number of items per questionnaire (Supporting Information S3).

To cluster the compressed data, we initially identified the number of clusters (=*k*) by using the full cohort and applying the K‐means algorithm with 50 different initial conditions (five random seeds for 10 imputations). Using this prior knowledge, we then performed consensus clustering. For this, we repeatedly used the K‐means algorithm on 500 randomly drawn subsets of the data (50 random seeds for 10 imputations) that included only 60% of the individuals and searched for *k* clusters. We set the conclusive cluster based on the consensus across repetitions (i.e., individuals who repeatedly clustered together received the same conclusive cluster label). Individuals with extraordinarily low consensus with their clusters were excluded from further analyses (*n* = 16).

### 2.5 Identification of Most Differentiating Features

To explain the differences between the clusters, we performed statistical tests on the data used in the clustering process. This analysis focused on the differences between the clusters that mainly contained individuals from the NBL since this was the group of interest.

Depending on the type of each variable, we calculated an appropriate effect size and set conservative thresholds to categorise them as either strongly differentiating or not differentiating. For intervals and ratios, we calculated the standardised effect size using Cohen's *d* with pooled variance for groups with unequal variance and unequal sample sizes (*d* ≥ 0.8 and *d* ≤ 0.2 for strongly diff. and not diff.). For ordinal categories, we calculated the odds ratio using ordinal logistic regression (OR > 4 and OR < 2 for strongly diff. and not diff.; applying the reciprocal). For nominal data with more than two categories, we calculated Carmér's V using chi‐square (V > 0.5 and V < 0.1 for strongly diff. and not diff.). For nominal data with two categories, we calculated the odds ratio using Fisher's exact test (OR > 10 and OR < 2 for strongly diff. and not diff.; applying the reciprocal).

### 2.6 Post Hoc Analysis

To explore further differences between the identified clusters, we analysed variables that were not used for clustering. Specifically, we assessed the impact of psychiatric comorbidities, established NT1 biomarkers and objective sleep measures.

The impact of psychiatric symptoms and comorbidities was assessed using data derived from either the MINI, the HADS or the BDI (Sheehan et al. 1998; Zigmond and Snaith 1983). These questionnaires regarding psychiatric comorbidities were previously excluded from the clustering because, after the internationalisation of the iSPHYNCS, the study participants received only a subset of these questionnaires due to changes in the study protocol. Therefore, if we included them in the clustering pipeline, participants might have clustered based on the questionnaires they received and not on the responses.

Where available, hypocretin‐1 levels and human leukocyte antigen (HLA) DQB1*0602 phenotyping were included, as these are established biomarkers for NT1 (Bassetti et al. 2019). Regarding objective sleep measures, we compared the mean sleep latency from the multiple sleep latency test (MSLT) (Littner et al. 2005) between the clusters. In addition, data from physical activity recordings (Fitbit Inspire HR/2/3) worn by participants over the course of up to 1 year were analysed to extract sleep parameters such as sleep duration and bedtime/get‐up time. Participants were instructed to wear the Fitbit on the non‐dominant arm. These data were acquired using a custom pipeline (Gnarra et al. 2024).

The normality of distributions was assessed using Shapiro–Wilk tests. For normally distributed data, *p*‐values were calculated using Welch's *t*‐test for unequal variances. For non‐normally distributed data, *p*‐values were derived from Mann–Whitney U tests.

### 2.7 Ethical Approval

The study protocol received ethical approval from the relevant authorities in each participating country, and all participants provided informed consent in accordance with the Declaration of Helsinki. Ethical approval for the study was obtained from local ethics committees (Ethics Committees ID: 2019–00788 in Switzerland, 202/2022 in Germany, and NL84710.058.23 in the Netherlands), and the trial is registered in the international clinical trials registry (ClinicalTrials.gov Identifier: NCT04330963).

## 3 Results

### 3.1 Population

Originally, 260 participants were included in the iSPHYNCS cohort as of October 2024. Of these, 40 participants were excluded: 39 due to insufficient data and one due to a hypersomnia diagnosis that was ultimately attributed to a systemic disease, which constituted an exclusion criterion. Thus, the final analysis included 220 participants, whose baseline characteristics are summarised in Table 1.

**TABLE 1:** Baseline demographics.

	Missing	Overall	Healthy controls	Narcolepsy type 1	Narcolepsy borderland
*n*		220	33	51	136
Age, median [Q1, Q3]	0	26 [21, 33]	28 [24, 32]	26 [21, 35]	26 [21, 33]
BMI, median [Q1, Q3]	5	23.6 [21.1, 27.2]	22.9 [21.3, 25.8]	23.9 [21.9, 28.5]	23.7 [20.7, 27.5]
Gender (f), *n* (%)	0	155 (70.5)	20 (60.6)	30 (58.8)	105 (77.2)
Work active, *n* (%)	12	148 (71.2)	26 (81.2)	27 (55.1)	95 (74.8)
Education, *n* (%)	7				
Secondary		43 (20.2)	0 (0.0)	12 (23.5)	31 (23.8)
Apprenticeship		73 (34.3)	6 (18.8)	17 (33.3)	50 (38.5)
High school		36 (16.9)	8 (25.0)	9 (17.6)	19 (14.6)
University		58 (27.2)	17 (53.1)	13 (25.5)	28 (21.5)
Other		3 (1.4)	1 (3.1)	0 (0.0)	2 (1.5)
Hypocretin, median [Q1, Q3]	133	270 [88,320]	na	44 [19.5,88]	299 [273,333]
HLA:DQB1*06.02 positive, *n* (%)	73	54 (36.7)	3 (20.0)	31 (96.9)	20 (20.0)
Medication, *n* (%)	0				
No medication		116 (52.7)	33 (100.0)	13 (25.5)	70 (51.5)
Antidepressant		21 (9.5)	0 (0.0)	3 (5.9)	18 (13.2)
Sodium Oxybate		3 (1.4)	0 (0.0)	2 (3.9)	1 (0.7)
Stimulant		66 (30.0)	0 (0.0)	32 (62.7)	34 (25.0)
Other		14 (6.4)	0 (0.0)	1 (2.0)	13 (9.6)

The median age of participants was 26 years (interquartile range [IQR]: 21–33), and 70.5% were female. The cohort consisted of 51 participants with NT1, 33 HC, and 136 participants in the NBL. Most participants were employed or studying, with 71.2% reporting active engagement in work or education, although this was lower in the NT1 group (55.1%) compared to HCs (81.2%) and the NBL group (74.8%).

### 3.2 Cluster Analysis

**FIGURE 2:**
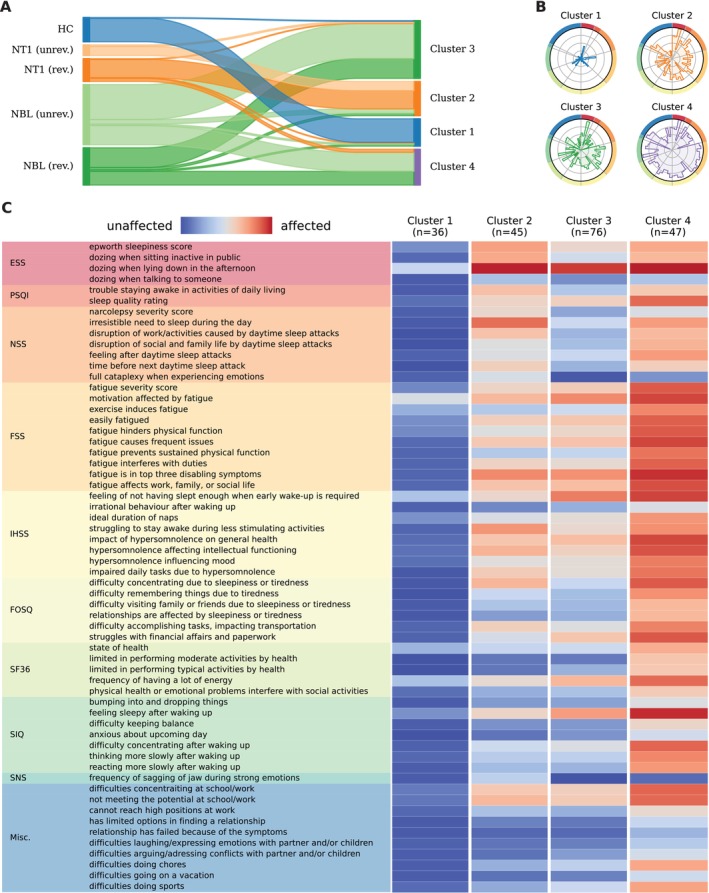
Description of the four identified clusters. (A) The flowchart shows how the study cohorts are distributed into the clusters. The patient groups are further divided if the diagnosis are reviewed or not (rev. = reviewed diagnosis, unrev. = unreviewed diagnosis). (B) The radar plots highlight how the clusters have unique footprints regarding the perceived affectedness (see C for the colour‐coded questions). Cluster 1 is virtually unaffected, clusters 2 and 3 are mild phenotypes that differ in the affectedness regarding cataplexy, and cluster 4 reports overall high affectedness. (C) The barcode plot provides a description of the most differentiating features. The colour code from blue to red corresponds to the magnitude of the radar plots in B. ESS, Epworth Sleepiness Scale; FOSQ, Functional Outcome of Sleep Questionnaire; FSS, Fatigue Severity Scale; HC, Healthy controls; IHSS, Idiopathic Hypersomnia Severity Scale; Misc., Miscellaneous; NBL, Narcolepsy Borderland; NSS, Narcolepsy Severity Scale; NT1, Narcolepsy Type 1; PSQI, Pittsburgh Sleep Quality Index; SF36, Short Form‐36; SIQ, Sleep Inertia Questionnaire; SNS, Swiss Narcolepsy Scale.

The consensus clustering resulted in four distinct clusters, as illustrated in Figure 2. Below, we describe the characteristics of each cluster in detail.

#### 3.2.1 Cluster 1

This cluster (*n* = 36) included participants who were largely unaffected across all domains analysed. Nearly all HC (*n* = 31) fell into this group, with only one excluded due to low consensus and another being in a different cluster. Both expressed higher levels of anxiety and pain. Additionally, this cluster included four individuals from the NBL and one patient with NT1, reflecting a general absence of significant symptoms.

#### 3.2.2 Cluster 2

This cluster (*n* = 45) was primarily characterised by pronounced daytime sleepiness and a higher likelihood of experiencing cataplexy. Participants frequently reported emotion‐triggered muscle weakness and an increased propensity to fall asleep during the day. In contrast, compared to cluster 4, these individuals reported lower levels of fatigue and fewer restrictions in daily life. This cluster mainly contains patients categorised as NT1 and 10 patients from the NBL.

#### 3.2.3 Cluster 3

This cluster (*n* = 76) included participants predominantly from the NBL exhibiting a milder phenotype. Compared to cluster 4, patients in cluster 3 reported fewer restrictions in daily activities, better sleep quality, and an absence of emotion‐triggered muscle weakness (Figure 2, Table S1). Notably, gender, medication use, questions from the PSQI and morningness‐eveningness preferences did not significantly differentiate between this group and cluster 4 (Table S2). Three patients with NT1 and one healthy control were assigned to this cluster.

#### 3.2.4 Cluster 4

This cluster (*n* = 47) comprised predominantly participants from the NBL with a more severe symptom profile. It also included four individuals with an NT1 diagnosis. Participants in this group exhibited significantly higher levels of fatigue, with both cognitive and physical components of fatigue contributing to increased exhaustibility. Additionally, this cluster was characterised by excessive daytime sleepiness, sleep drunkenness, poor sleep quality, and a pronounced sense of restriction and underachievement in daily activities. Individuals in this group reported substantial functional impairments across multiple domains, including cognition, social interactions, and workplace or academic performance.

Comprehensive tables for all differentiating features are provided in the Supporting Information S4.

### 3.3 Individuals With Unexpected Cluster Assignment

There were eight individuals with an NT1 diagnosis who were assigned to different clusters than cluster 2, which contained the majority of NT1 patients. In cluster 4, there were four (8.5%) participants with a diagnosis of NT1 (three definite with low hypocretin levels, one probable) who reported enhanced fatigue, sleep drunkenness, and impairment in daily activities.

Three individuals with NT1 diagnoses were assigned to cluster 3 (two definite, one probable), and another with a confirmed NT1 diagnosis and low hypocretin levels was placed in cluster 1. All four individuals were receiving stimulant treatment, suggesting either fluctuating or milder phenotypes influenced by medication effects. Additionally, four participants from the NBL were assigned to cluster 1 due to a less affected phenotype. Notably, only one of these participants was taking medication at the time of assessment.

Furthermore, 10 patients from the NBL were assigned to cluster 2, primarily because they reported muscle weaknesses triggered by emotions but did not report the pronounced fatigue observed in cluster 4. There was one healthy control in cluster 3 that reported higher levels of pain and anxiety. Proportionally, no significant centre differences were observed among individuals with unexpected cluster assignment.

### 3.4 Post Hoc Analysis

**FIGURE 3:**
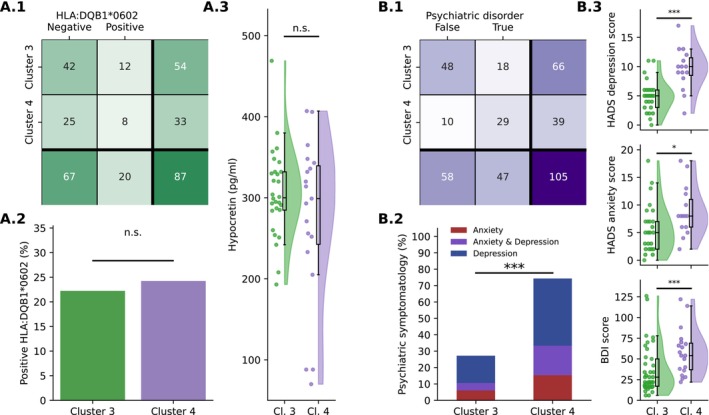
Comparison of NT1 biomarkers and psychiatric disorders between cluster 3 and cluster 4. (A) (1) Contingency table of the HLA:DQB1*0602 positivity. The total number of individuals was lower since the analysis was not available for every individual (43/61 and 46/71). (2) The barplot with the proportion of HLA:DQB1*0602 positive individuals shows that there is no significant difference between the clusters. (3) The hypocretin levels were not significantly different between the clusters. (B) (1) Contingency table showing the presence of psychiatric symptomatology based on the HADS and MINI questionnaires. The total number of individuals was lower since not every individual provided answers to the questionnaires (54/61 and 60/71). (2) The barplot shows that cluster 4 had a significantly higher proportion of individuals with a psychiatric symptomatology. (3) Cluster 4 scored higher in the HADS questionnaire for depression and also in the BDI compared to cluster 3.

To better characterise the difference between the two borderland clusters, we evaluated the biomarkers HLA:DQB1*0602 and hypocretin‐1 levels, as well as the burden of psychiatric symptoms and comorbidities using validated questionnaires (Figure 3).

There was no significant difference regarding HLA:DQB1*0602 positivity (22.2% vs. 24.2%, *p* = 1.0). Moreover, there was no significant difference between the groups regarding the hypocretin‐1 levels (305 ± 54 vs. 275 ± 101, *p* = 0.625). Most participants (105/123, 85.4%) had either results from the MINI, BDI, or the HADS score to calculate the burden of psychiatric symptomatology. Cluster 4 had a significantly higher prevalence of either anxiety or depression than cluster 3 (74.4% vs. 27.3%, *p* < 0.001), with a higher mean HADS depression score (9.60 ± 3.60 vs. 4.96 ± 2.75, *p* < 0.001), HADS anxiety score (8.87 ± 4.50 vs. 5.84 ± 4.45, *p* = 0.030) and BDI score (61.7 ± 32.0 vs. 34.2 ± 27.9, *p* < 0.001).

**FIGURE 4:**
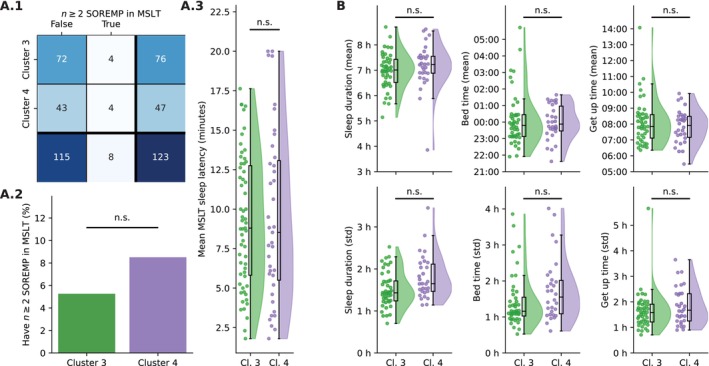
Objective sleep measures. (A) Sleep parameters from MSLT. There were no significant differences between the clusters regarding the number of SOREMPs and the mean MSLT sleep latency. (B) Sleep parameters from long‐term physical activity recordings. There were no significant differences, although it seems like cluster 4 demonstrates a higher variability in sleep duration and bedtime, indicating poorer sleep hygiene.

There was no significant difference between cluster 3 and cluster 4 in the REM propensity (5.3% vs. 8.5%, *p* = 0.480) nor in the mean sleep latency of the MSLT (9.2 ± 4.2 vs. 9.5 ± 5.4, *p* = 0.919), as shown in Figure 4A. Although not statistically significant, it seems like cluster 4 demonstrates a higher variability in sleep duration (108 ± 30 min vs. 91 ± 25 min, *p* = 0.078) and bedtime (105 ± 51 min vs. 85 ± 43 min, *p* = 0.300), indicating poorer sleep hygiene (Figure 4B).

## 4 Discussion

This study aimed to examine the clinical heterogeneity of central disorders of hypersomnolence (CDH) by employing unsupervised clustering methods to identify distinct phenotypic subgroups. It is the first study to develop a computational pipeline powerful enough to differentiate between groups by utilising only readily available data from questionnaires. The findings highlight the diverse symptom profiles among individuals with narcolepsy and its borderland, including, for the first time, healthy controls, emphasising the utility of clustering in refining diagnostic and therapeutic approaches.

The identification of clusters underscores the clinical variability within the CDH spectrum. Cluster 4 represents a subgroup with questionnaire data pointing towards severe functional impairments and morbidity, as evidenced by high levels of cognitive and physical fatigue, sleep drunkenness and low sleep quality. Daily activities, including work, school and social interactions, as well as cognitive functions (e.g., remembering, concentrating), seem significantly affected. Cluster 4 is the only cluster also showing increased physical exhaustibility (e.g., FSS: ‘exercise induces fatigue’, ‘fatigue prevents sustained physical function’). These features, although described in CDH (Droogleever Fortuyn et al. 2012), are more commonly observed in affective disorders and other fatigue‐related conditions (e.g., post‐infectious fatigue, chronic fatigue syndrome). Notably, this cluster included mainly individuals from the narcolepsy borderland but also a few definite NT1 (low hypocretin). While previous literature has often emphasized NT1 as the most severe form of hypersomnolence, our findings reveal that a substantial proportion of NBL patients also experience profound functional impairment. The smaller proportion of NT1 in this cluster could reflect variability in symptom expression or treatment effects. These findings suggest that severe functional impairments may overshadow specific biomarkers, including hypocretin deficiency. Cluster 4 may represent patients characterised by significant morbidity likely requiring more intensive clinical management (Barateau et al. 2017; Vernet et al. 2010). Affective disorders should be optimally treated, if necessary pharmaceutically (Barateau et al. 2017, 2020). The pattern and severity of complaints (e.g., fatigue, exhaustibility) might also require some additional and tailored therapeutic strategies, like psychotherapy, physiotherapy and pacing strategies.

Cluster 3, in contrast, represents a milder phenotype, with less impact on daily functioning, suggesting a distinct pathophysiological or psychosocial trajectory compared to Cluster 4. Supporting those first findings, the post hoc analyses revealed significant differences in psychiatric comorbidity between the two borderland clusters. Cluster 4 had a notably higher prevalence of anxiety or depression compared to Cluster 3 (74.4% vs. 27.3%), confirmed by elevated scores on validated psychiatric scales such as the HADS and BDI. This aligns with previous literature suggesting a strong association and probably bi‐directional relationship between hypersomnolence and mood disorders, particularly in individuals with greater functional impairment (Aktan Suzgun et al. 2024; Lopez et al. 2017; Huang et al. 2025).

Cluster 2 was characterised by pronounced cataplexy, excessive daytime sleepiness, and increased susceptibility to unintentional daytime sleep episodes, closely aligning with the clinical presentation of narcolepsy type 1 (Bassetti et al. 2019). This cluster reinforces the diagnostic relevance of cataplexy as a key distinguishing feature among CDH subgroups (Zhang et al. 2018). However, the assignment of individuals from the NBL to this cluster suggests potential challenges in accurately interpreting emotion‐triggered muscle weakness. This finding highlights the importance of conducting a comprehensive clinical interview to differentiate cataplexy from its mimics (Bassetti et al. 2019).

Finally, Cluster 1 included predominantly healthy controls, providing a useful baseline for comparison and further emphasising the phenotypic differences across groups.

The assignment of eight individuals with a NT1 diagnosis to clusters outside the expected cluster 2 highlights the variability in symptom expression. Cluster 4, with its emphasis on severe fatigue, sleep drunkenness, and functional impairments, included three individuals with definite NT1 and low hypocretin levels, as well as several participants with incomplete diagnostic profiles (Trotti 2017). These findings underscore the importance of considering atypical presentations in NT1, psychiatric comorbidities, as well as the potential influence of factors such as medication, fluctuating symptoms, or incomplete data (e.g., missing hypocretin‐1 or HLA‐DQB1 measurements) (Maski et al. 2017; Ozaki et al. 2012).

Interestingly, the presence of participants with NT1 diagnoses in clusters 3 and 1 suggests the existence of milder or fluctuating phenotypes within the disorder (Gool et al. 2022). This could reflect the dynamic nature of symptom expression in NT1, particularly in cases where medication or lifestyle modifications may reduce symptom burden (Becker et al. 2004; Bogan et al. 2017; Emsellem et al. 2020). Furthermore, the assignment of four participants from the NBL to the healthy cluster (cluster 1) reinforces the concept that the borderland encompasses a spectrum of severity, with some individuals exhibiting minimal functional impairment (Baumann et al. 2014; Nevsimalova et al. 2021).

Interestingly, no significant differences were observed in HLA‐DQB1 positivity between the two borderland clusters, suggesting that HLA‐associated genetic predisposition does not account for the observed disparities. Furthermore, our MSLT analysis showed no significant differences in SOREMPs between the two clusters, aligning with previous findings that REM propensity was not a key differentiating factor (Gool et al. 2022). Instead, environmental, behavioural, or other genetic factors may play a more prominent role in shaping the clinical phenotype (Ohayon 2013; Ruoff et al. 2017).

Although statistically not significant, data from wearable devices further hinted at potential differences between clusters 3 and 4 in terms of sleep hygiene. Participants in cluster 4 exhibited more variable sleep durations, as well as irregular bedtimes and wake times, indicative of poorer sleep hygiene. This aligns with previous findings, where differences in sleep duration between weekdays and weekends were important for clustering (Gool et al. 2022). Such patterns can be indicative of psychiatric comorbidities and may additionally contribute to the greater functional impairment observed in this group. Furthermore, it may represent a modifiable target for interventions aimed at improving daytime functioning (Tadrous et al. 2021; Parmar et al. 2019).

The cluster‐based approach highlights phenotypic variability that is not fully captured by current diagnostic criteria for NT1 and narcolepsy borderland (Fronczek et al. 2020). These findings underscore the need for a more dynamic diagnostic framework that incorporates both clinical and biomarker variability (Gauld et al. 2021). While current criteria rely heavily on features of the multiple sleep latency test, our results suggest that functional impairment and psychiatric comorbidities may also play a critical role in phenotypic differentiation (Trotti et al. 2013; Dietmann, Gallino, Wenz, et al. 2021). For instance, participants in cluster 4 with a narcolepsy borderland diagnosis exhibited a symptom burden comparable to, sometimes even exceeding, patients with NT1. This finding underscores the clinical overlap that can occur between different central disorders of hypersomnolence in real‐world practice, particularly when considering functional impairment, fatigue, and psychiatric comorbidity. Such overlap may challenge the practical application of strictly categorical diagnostic criteria and suggests that complementary frameworks could help capture the full range of clinical presentations.

It is important to note that prior CDH studies historically have restricted patient inclusion to conform to existing classification criteria (i.e., ICSD3) (Gool et al. 2022). The inclusion criteria of the iSPHYNCS observational study are relatively broad which, we feel, better reflects real‐world patient populations. Specifically, iSPHYNCS only defines two groups of included participants, i.e., NT1 and healthy controls. All other patients with disorders from the NBL only require a subjective complaint of excessive daytime sleepiness and the exclusion of severe co‐occurring comorbidities providing a clear explanation for the patients' symptoms. Patients with comorbid affective disorders may be included, but only if the degree of excessive sleepiness as viewed by the study physician cannot be explained by the comorbid psychiatric condition. The iSPHYNCS approach suggests that psychiatric comorbidities may play an important role in patients who typically present to a sleep disorders clinic. Finally, to what extent Cluster 3 and Cluster 4 may be further divided into additional clusters based on other biomarkers such as microbiome, long‐term Fitbit activity, proteomics or other genetic analyses, as are planned for the iSPHYNCS study, remains to be determined. Further work is planned to better understand how patients diagnosed with NT2 or idiopathic hypersomnia based on existing ICSD3 criteria may cluster as the iSPHYNCS multimodal analyses are developed.

This study has several limitations that should be acknowledged. First, while the clustering approach provided meaningful subgroup differentiation, the reliance on self‐reported questionnaires introduces the potential for recall bias and subjective variability in symptom reporting. Second, although we included a relatively large sample from multiple centres, the generalizability of our findings may be limited by demographic and clinical differences across study sites. Third, the absence of objective biomarkers such as hypocretin‐1 levels for all participants restricts definitive diagnostic confirmation, particularly for individuals with NT1. Additionally, medication effects were not fully controlled, which may have influenced symptom severity and cluster assignment. Fourth, although participants were instructed to complete questionnaires based on their status prior to treatment initiation, medication effects cannot be fully excluded. Furthermore, psychiatric comorbidity data were excluded from the clustering analyses, as only a subset of participants received these questionnaires due to protocol changes, which could have introduced bias based on questionnaire availability rather than participants' characteristics. Finally, while our clustering method demonstrated robustness, alternative machine learning approaches could yield different subgroups, warranting further validation in independent cohorts.

### 4.1 Broader Clinical and Research Implications

The identification of distinct phenotypic clusters has several important implications for clinical practice and research:

1. *Personalised Management:* The variability in symptom burden, psychiatric comorbidities, and sleep hygiene across clusters highlights the need for individualised treatment approaches. For example, participants in cluster 4 may benefit from interventions targeting fatigue and psychiatric symptoms, while those in cluster 3 may require less intensive management.
2. *Refining Diagnostic Understanding:* The overlap of some symptoms between NT1 and narcolepsy borderland, as well as the presence of patients with NT1 in multiple clusters, suggests that current diagnostic criteria may not fully capture clinical variability. Incorporating cluster‐based phenotyping could complement traditional diagnostic approaches by highlighting patterns that may inform prognosis or guide individualised care strategies. At present, such approaches should be viewed as exploratory and potentially useful for screening or stratification, particularly in settings where access to specialised diagnostic testing is limited.
3. *Future Research Directions:* Longitudinal studies are needed to assess the stability of cluster assignments over time and to determine whether cluster affiliation predicts clinical outcomes or treatment response. Additionally, further exploration of the underlying mechanisms driving differences between clusters—such as genetic, biological, lifestyle, or environmental factors—could yield new insights into the pathophysiology of central disorders of hypersomnolence.

## 5 Conclusions

This study demonstrates the utility of cluster analysis using only questionnaires and easily available metadata in uncovering phenotypic heterogeneity within CDH, challenging traditional diagnostic boundaries and highlighting the need for a more individualised approach to diagnosis and management. By integrating clinical, biomarker, and wearable device data, future research can build on these findings to refine diagnostic frameworks and allow tailored therapies for individuals across the CDH spectrum.

## Author Contributions

**Rafael Morand:** conceptualization, methodology, software, formal analysis, visualization, writing – original draft, writing – review and editing. **Livia Fregolente:** conceptualization, data curation, investigation, formal analysis, writing – original draft, writing – review and editing. **Julia van der Meer:** data curation, methodology, writing – review and editing. **Elena S. Wenz:** investigation, data curation, writing – review and editing. **Annina Helmy:** methodology, software, writing – review and editing. **Lorenzo Brigato:** methodology, software. **Jan D. Warncke:** investigation, data curation, writing – review and editing. **Kseniia Zub:** investigation, data curation, writing – review and editing. **Ramin Khatami:** investigation, methodology, writing – review and editing. **Zhongxing Zhang:** methodology, data interpretation, writing – review and editing. **Sigrid von Manitius:** investigation, writing – review and editing. **Silvia Miano:** investigation, writing – review and editing. **Jens Acker:** investigation, writing – review and editing. **Mathias Strub:** investigation, writing – review and editing. **Ulf Kallweit:** investigation, writing – review and editing. **Gert Jan Lammers:** investigation, writing – review and editing. **Athina Tzovara:** methodology, writing – review and editing. **Claudio L. A. Bassetti:** funding acquisition, resources, project administration, writing – review and editing. **Stavroula Mougiakakou:** conceptualization, supervision, methodology, supervision, writing – review and editing. **Markus H. Schmidt:** conceptualization, supervision, project administration, writing – review and editing. All authors critically revised the manuscript and approved the final version.

## Funding

iSPHYNCS is an investigator‐initiated research project. This project is financially supported by two Swiss National Science Foundation project grants (SNSF Grant/Award Number: 320030_185362 and 3203B_215721), and by the Direktion Lehre und Forschung (DLF) Inselspital Bern Biobank Call 2017.

## Ethics Statement

The study was approved by local ethical committees (Ethics Committees ID: 2019–00788 in Switzerland, 202/2022 in Germany, and NL84710.058.23 in the Netherlands).

## Consent

Each patient was enrolled to the study after an informed consent.

## Conflicts of Interest

The authors declare no conflicts of interest.

## Supporting information


**Data S1:** Supporting Information.

## Data Availability

The data that support the findings of this study are available on request from the corresponding author. The data are not publicly available due to privacy or ethical restrictions.
